# A literature review and case report of severe and refractory post-colectomy enteritis

**DOI:** 10.1186/s12876-019-0974-4

**Published:** 2019-04-25

**Authors:** Yingyun Yang, Yuan Liu, Weiyang Zheng, Weixun Zhou, Bin Wu, Xiyu Sun, Wei Chen, Tao Guo, Xiaoqing Li, Hong Yang, Jiaming Qian, Yue Li

**Affiliations:** 1 Department of Gastroenterology, Peking Union Medical College Hospital, Chinese Academy Medical Sciences and Peking Union Medical College, No. 1 Dongshuaifuyuan, Dongcheng District, Beijing, 100730 China; 2 Department of Pathology, Peking Union Medical College Hospital, Chinese Academy Medical Sciences and Peking Union Medical College, Beijing, China; 3 Department of General Surgery, Peking Union Medical College Hospital, Chinese Academy Medical Sciences and Peking Union Medical College, Beijing, China; 4 Department of Nutrition, Peking Union Medical College Hospital, Chinese Academy Medical Sciences and Peking Union Medical College, Beijing, China

**Keywords:** Ulcerative colitis, IPAA, Post-colectomy enteritis

## Abstract

**Background:**

Ulcerative colitis (UC)-related post-colectomy enteritis is a very rare condition that is characterized by diffuse small-bowel mucosal inflammation following colectomy and could be very dangerous. In previously reported cases, corticosteroid therapy seemed to be the optimal choice for inducing remission; however, the patient studied herein presented with severe diarrhoea and hypovolemic shock and failed to achieve full remission with corticosteroid therapy.

**Case presentation:**

We describe the case of a patient with severe pan-enteritis presenting with life-threatening diarrhoea complicated with hypovolemic shock and acute kidney injury after colectomy and ileal pouch anal anastomosis (IPAA) for UC; this patient was successfully treated by ileostomy closure after failing to achieve full remission with corticosteroid therapy. Next, we review other cases of post-colectomy enteritis reported in the literature and propose a flow-chart for its diagnosis and initial treatment.

**Conclusion:**

Post-colectomy enteritis can be dangerous, and the early awareness of this condition plays a vital role. Additionally, in patients who do not respond well to corticosteroid or immunosuppressant therapy, early closure of the ileostomy and re-establishment of the natural faecal stream could be important considerations.

**Electronic supplementary material:**

The online version of this article (10.1186/s12876-019-0974-4) contains supplementary material, which is available to authorized users.

## Background

Ulcerative colitis (UC) has been characterized by superficial and diffuse inflammation limited to the colon and rectum. Usually, small-bowel involvement in UC manifests as backwash ileitis or post-colectomy pouchitis [1]. UC-related post-colectomy enteritis is characterized by diffuse small-bowel mucosal inflammation following colectomy [2, 3].

In previously reported cases, corticosteroid therapy seemed to be the optimal choice for inducing remission [2]. However, in this report, we describe the case of a patient with post-colectomy enteritis who presented with severe diarrhoea and hypovolemic shock and failed to achieve full remission with corticosteroid therapy but fully recovered after ileostomy closure. Additionally, we review the relevant cases in the literature and propose a diagnostic algorithm and treatment strategy.

## Case presentation

**Fig. 1:**
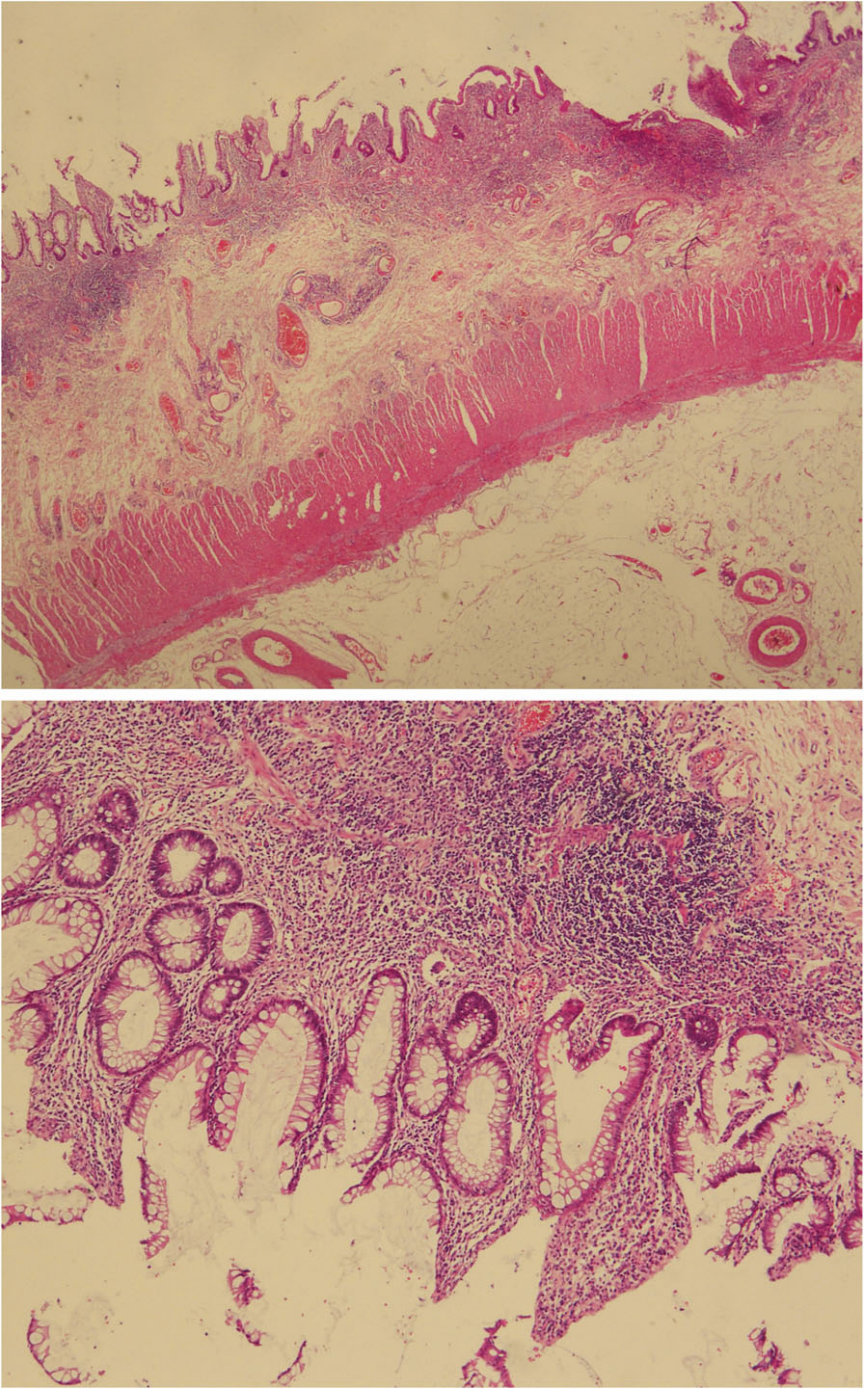
Pathological images of the resected colon specimen. Acute and chronic inflammation are visible in the mucosal and submucosal layers. Multifocal ulcers and crypt abscesses are visible, which are consistent with the diagnosis of UC (× 100, × 200)

A 51-year-old woman who presented with diarrhoea containing mucus and blood had initially been diagnosed with acute severe ulcerative pan-colitis and backwash ileitis at the age of 49 years at Peking Union Medical College Hospital (PUMCH) in December 2014. She had poliomyelitis when she was very young, and there was nothing special regarding her family or psychosocial history. Serology was positive for perinuclear antineutrophil cytoplasmic antibody (pANCA) and negative for anti-*Saccharomyces cerevisiae* antibody (ASCA). Her condition was refractory to steroids and complicated by Cytomegalovirus (CMV) infection. Ultimately, she underwent sub-total colectomy and ileostomy in February 2015. Pathological examination of the resection specimen showed diffuse pan-colitis consistent with UC and no indications of Crohn’s disease (Fig. 1). She did well in the following 11 months; prednisone was tapered and stopped within 2 months, and she gained 5 kg of weight after the ileostomy. In January 2016, a scheduled restorative ileal pouch-anal anastomosis (IPAA) with proximal neo-ileostomy was performed.

**Fig. 2:**
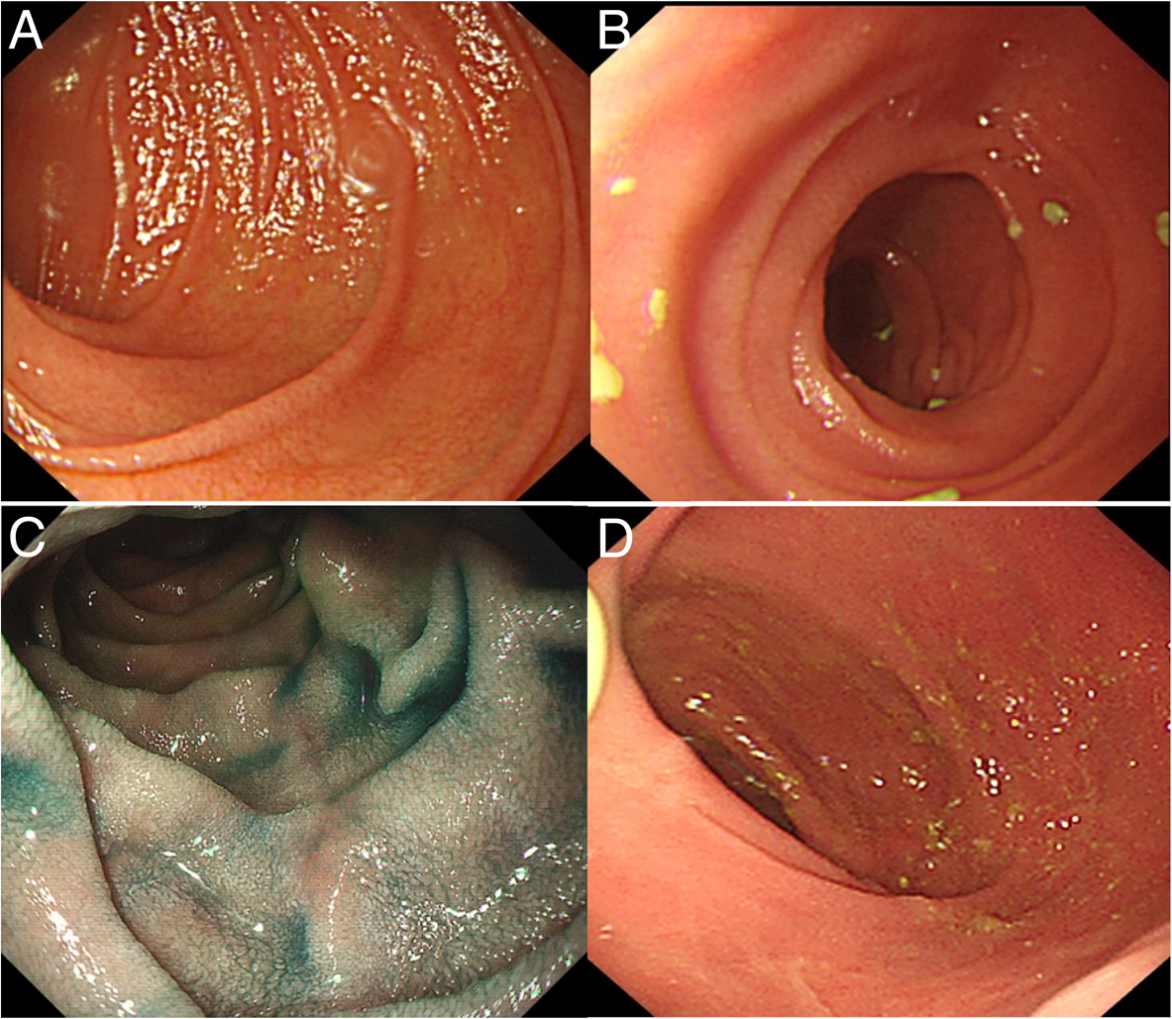
Endoscopic images: **a**, **b** at the onset of enteritis; (**c**, **d**) at the last follow-up in March 2018. **a** Gastroscopy showing a normal descending duodenum and blunt intestinal villi. **b** Endoscopy through the loop ileostomy showing the normal appearance of the neo-ileum. **c** Gastroscopy with indigo carmine staining showing recovery of the villous atrophy. **d** Pouchoscopy revealing the normal appearance of the neo-ileum in March 2018

**Fig. 3:**
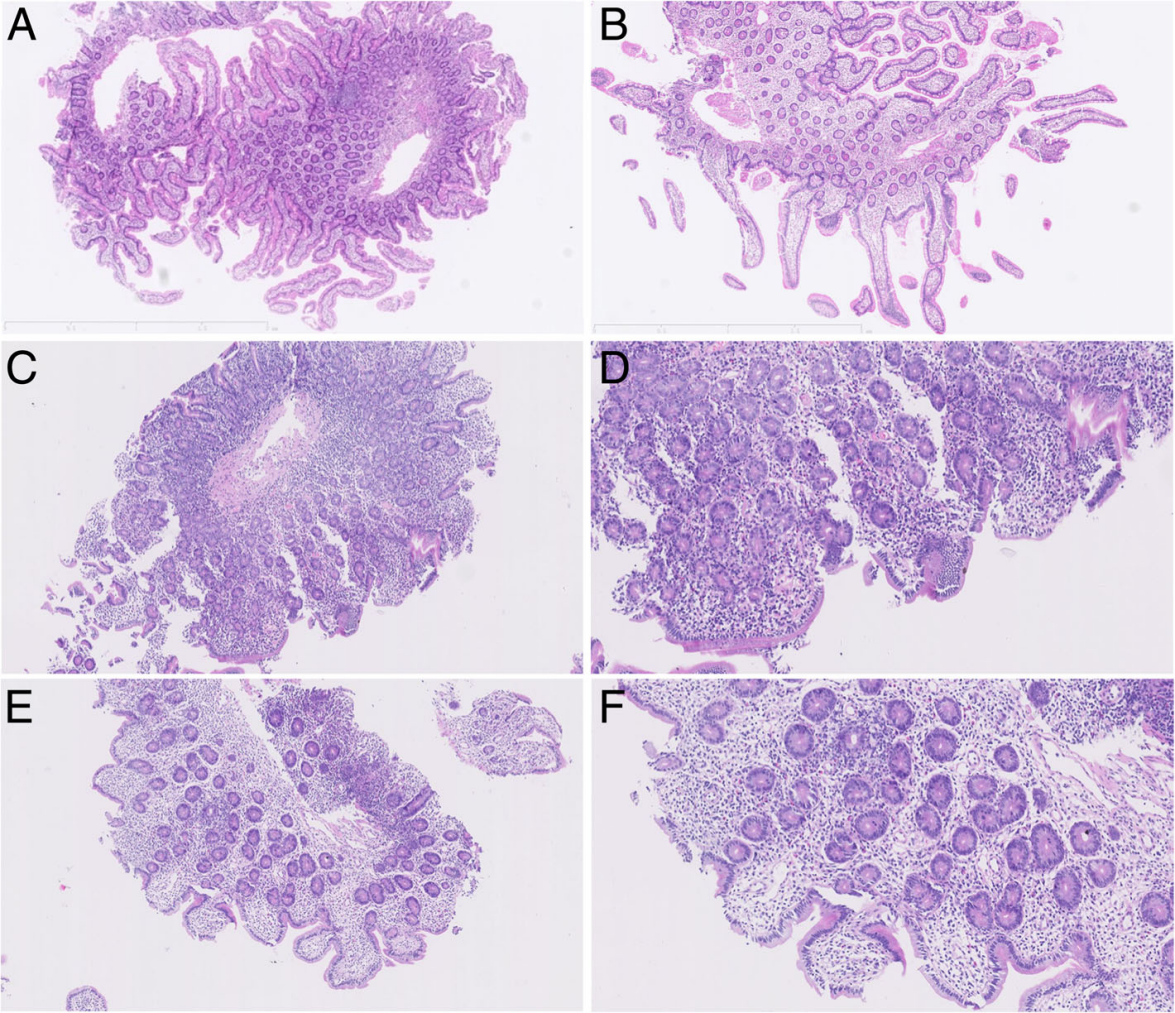
Pathological findings: **a**-**d** at the onset of enteritis; (**e**-**f**) at the last follow-up in March 2018. **a**, **b** Pathological images of the duodenum at the onset of enteritis (× 100, × 200): acute and chronic inflammation with some cryptitis, few goblet cells and moderate villous atrophy. **c**, **d** Pathological images of the neo-ileum through the ileostomy at the onset of enteritis (× 100, × 200): acute and chronic inflammation with cryptitis, some irregular crypt structures, mild villous atrophy, few goblet cells. **e**-**f** Pathological images of the duodenum (**e**) and neo-ileum (**f**) at the last follow-up: recovery of the chronic inflammation, number of goblet cells and villous structures; cryptitis is not observed

From one month after the IPAA, her 24-h stool collection slowly increased to 1.5–2 L. Next, she noticed decreased urine output since April. In early May 2016, she presented to our emergency room with repeated unconsciousness over the course of 10 days. Her vital signs were as follows: blood pressure (BP), 74/50 mmHg; heart rate (HR), 90 bpm; additionally, she exhibited a poor nutritional status (160 cm; 39 kg). On physical examination, active bowel sounds were noticed to occur approximately 7–9 times per minute. Her serum creatinine level was 183 μmol/L, indicating acute kidney injury. Treatment with fluid replacement and noradrenaline maintained her BP at 80–90/50–60 mmHg and gradually normalized her creatinine level. However, her 24-h watery stool collection persisted, and she developed fever and vomiting. While many leukocytes were found in stool collected from the diverted ileostomy, repeated stool cultures and tests for *Clostridium difficile* toxins were negative. Tests for CMV-DNA, CMV-pp65 and EBV-DNA were performed and were all negative. The patient was not on any medications, including NSAIDs, upon verification. Her treatment with steroids was stopped before the end of April 2015. Empirical treatment with antibiotics, including ceftazidime, metronidazole and oral vancomycin, was administered with no response. Due to her reliance on noradrenaline, relative adrenal insufficiency was suspected, and hydrocortisone was initiated at 50 mg q6 h intravenously. Her stool volume decreased to less than 500 ml per day quickly, by which time the treatment with noradrenaline was successfully stopped. The levels of D-lactate, endotoxin and diamine oxidase indicated that the barrier function of the intestine was compromised and that bacterial translocation may have occurred. Oedematous inflamed mucosa with patchy superficial ulcers was observed in the diverted pouch by pouchoscopy. Although an upper endoscopy and an endoscopy through a stoma revealed a normal gross appearance in the stomach, duodenum and pre-stomal ileum (Fig. 2a, b), the histological examination of tissue biopsies of both the duodenum and pre-stomal ileum revealed enteritis, as indicated by moderate villous atrophy, cryptitis, decreased goblet cells, and severe active inflammation with neutrophil infiltration in the lamina propria, as well as negativity for intraepithelial lymphocytosis (Fig. 3a-d). From these lines of evidence of histological enteritis presenting in the duodenum, pre-stomal ileum and diverted pouch, we considered pan-enteritis to be present, and we diagnosed the patient with post-colectomy enteritis. The patient was treated with methylprednisolone at 30 mg intravenously once a day with tapering by 5 mg every 7 to 10 days; however, her stool volume from the ileostomy still gradually increased to 3–4 L. After a multidisciplinary team discussion, ileostomy closure was debated as the final rescue treatment and was performed in August 2016. Two months later, her stool volume decreased to less than 1 L per day, and she gained 2.5 kg of weight. Azathioprine at 50 mg/d was prescribed during the tapering of prednisone. Until the last follow-up in March 2018, she performed well, with an increase in body weight to 50 kg, and daily defecation approximately 5–6 times at less than 1 L/day, sometimes with form (Additional file 1). Gastroduodenal endoscopy and pouchoscopy were repeated annually and showed normal villi in the descending duodenum and neo-ileum in March 2018 (Fig. 2c, d). Gradually, the histology changed, showing recovery of the villous atrophy, cryptitis and inflammation in the lamina propria to normal (Fig. 3e-f).

## Discussion and conclusion

UC is normally restricted to the rectum and colon; however, exceptions include backwash ileitis, post-colectomy pouchitis, pre-stomal ileitis and post-colectomy enteritis [1, 4]. Post-colectomy enteritis, also called UC-related pan-enteritis, is characterized by diffuse, superficial, ulcerative mucosal inflammation of the entire small bowel, with a typical onset after colectomy for UC [4, 5].

We searched the PubMed, Medline and Embase databases for case reports and case series using the terms ‘ulcerative colitis’ and ‘post-colectomy enteritis’ or ‘pan-enteritis and included those published in English or with an English abstract before June 17, 2018. In addition to a literature review of 42 cases published in 2008, 11 other cases have been reported, including ours (Table 1). The median patient age was 43 years (range: 19–57 years). The symptoms included nausea, vomiting, abdominal pain, and a high ileostomy output [5, 6]. The differential diagnosis ranged from various infections to immune-mediated conditions, malignancies, ischaemia and toxic effects [7]. Endoscopy and pathology are essential to reveal the diffuse, superficial, and ulcerative mucosal inflammation in the small bowel, as in UC, and to differentiate them from the characteristics of Crohn’s disease, such as aphthous ulcers, deep ulcerations, strictures, skip lesions, and patchy and transmural inflammation [7, 8]. Although a secondary intestinal failure due to a long-term electrolyte and/or fluid deficit could not be ruled out completely, we believe that the diagnosis of post-colectomy pan-enteritis could be established based on the histological findings.

**Table 1:** Summary of cases of post-colectomy enteritis reported after 2008

No.	Sex	Age	Age at UC Dx	Extent of UC	Backwash ileitis	Serology	Surgery type	Onset of enteritis	Extent of enteritis	Infx	Induction therapy	Maintenance therapy	Remission	Procedure	FU
1 (this)	F	51	49	Pancolitis	No	pANCA+, ASCA-	1^st^ sub-TAC + ileostomy, 2^nd^ IPAA	4 mo	D, I, P	No	HC 50 mg q6 h → MP 30 mg qd iv	AZA (1.3 mg/kg/d)	AP	Closure of ileostomy	19 mo
2 [7]	M	23	17	Pancolitis	Yes	pANCA+, ASCA-	1^st^ TAC + ileostomy, 2^nd^ IPAA	3 mo	S, D, I, P	No	MP 40 mg qd iv (improved in 24 h)	AZA (3 mg/kg/d)	SS	Closure of ileostomy scheduled	10 mo
3 [7]	F	29	21	Pancolitis	No	ND	1^st^ TAC + ileostomy, 2^nd^ IPAA	4 mo	S, D, I, P	No	MP 40 mg qd iv (improved in 24 h)	AZA + allopurinol→certolizumab+MTX, P 10–20 mg	SD	NM	57 mo
4 [7]	M	30	2	Pancolitis	NM	pANCA+, ASCA-	TAC + IPAA	27 mo	S, D, I, P	No	MP 40 mg qd iv (improved in 24 h)	AZA (2 mg/kg/d)	SS	NM	43 mo
5 [7]	M	57	48	Pancolitis	No	pANCA+, ASCA-	TAC + ileostomy	27 mo	S, D, I	No	MP 40 mg qd iv (improved in 24 h)	AZA (2.2 mg/kg/d)	SS	NM	6 mo
6 [13]	F	43	37	Pancolitis	No	ANCA-, ASCA-	TAC + ileostomy	3 mo	S, D, I	No	MP 20 mg bid iv (did not improve)	Tacrolimus (po 3 mg bid, 4–8 ng/ml)	Since tacrolimus	NA	6 mo
7 [5]	M	56	46	Pancolitis	No	ND	TAC + IPAA	4 mo	D, I, P	No	HC 100 mg q6 h (improved in 48 h)	AZA (125 mg/d → stop	SS	NM	48 mo
8 [6]	F	56	44	Left-side	No	ND	Proctocolectomy+ileostomy	26 days	D, I, P	No	P 20 mg qd po → MP 20 mg tid iv	NA	Death	NA	Death
9 [14]	F	47	43	Pancolitis	NM	ND	colectomy+ ileostomy	4 mo	D, J	No	Corticosteroids (dose unknown)	Tacrolimus (po 3 mg bid)	SS	NM	NM
10 [11]	M	19	17	Pancolitis	NM	ND	proctocolectomy+IPAA	12 days	S, D, I	CMV	P 0.6 mg/kg/day po	IFX 5 mg/kg	AP	2nd op due to perforation	4 mo
11 [9]	F	25	25	21	Pancolitis	Yes	NM	9 days	D, J, I, P	No	P 40 mg qd po → DEX 8 mg qd	NA	SS	NM	NM

*FU* Follow-up, *NM* not mentioned, *ND* not detected, *NA* not applicable, *TAC* total abdominal colectomy,* IPAA* ileal pouch-anal anastomosis, *D* duodenum, *I* ileum, *P* pouch, *S* stomach, *J* jejunum, *Dx* diagnosis, *Infx* infection, *SD* steroid dependent, *AP* after procedure, *SS* since steroids

Seven previous case reports have described patients who developed enteritis within one month and whose conditions worsened quickly [3, 6, 9, 10]. In such cases, a delay in the diagnosis and the use of corticosteroids can be fatal (2/7 dead) or lead to dangerous conditions, such as acute perforation or massive bleeding (2/7) [6, 10, 11].

**Fig. 4:**
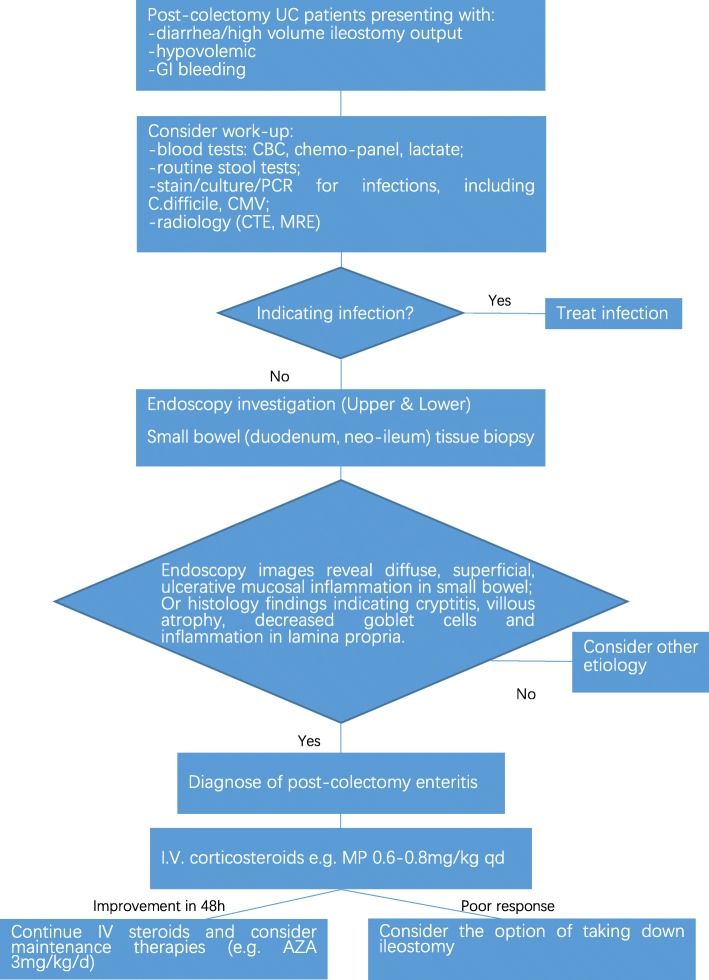
Proposed diagnostic algorithm and treatment strategy. Table 1 Summary of the cases of post-colectomy enteritis reported after 2008

Once the correct diagnosis is established, patients usually rapidly respond to treatment with intravenous corticosteroids and improve within 24 h [2, 7, 12]. Azathioprine, tacrolimus, and infliximab have been reported as maintenance therapies [11, 13, 14]. Only one case report described a scheduled loop ileostomy closure without describing the post-operative changes [7]. In our case, the use of hydrocortisone quickly improved our patient’s critical status. However, the symptoms did not fully resolve before the ileostomy was closed, indicating that patients may benefit from the re-establishment of a natural faecal stream. Thus, we propose a diagnostic algorithm and treatment strategy (Fig. 4). This algorithm includes a summary of experiences from previous reports in the literature [2, 7, 9]; in all cases, the patient underwent steroid treatment. The responses of six of nine patients whose steroid dosage ranged from 0.6 to 0.8 mg/kg/d were reported. Five patients showed significant improvement within 48 h. The sixth patient showed no improvement after steroid therapy, which is similar to the patient in our case [13]. In that case, the patient’s condition was relieved after treatment with tacrolimus. Unlike that patient, our patient achieved remission after closure of the ileostomy. Here, we propose a diagnostic algorithm and treatment strategy based on a few cases. An observational study with a larger number of cases will be extremely helpful for reaching a more precise conclusion. Unfortunately, post-colectomy enteritis in UC patients is a relatively rare condition. Thus, a study based on cases is much more feasible.

The pathogenesis of post-colectomy enteritis is unknown. There is a possibility that, in the setting of severe UC, the massive T cell- and cytokine-mediated inflammatory response may continue after colectomy with infiltration of the small bowel. Additionally, a change in faecal stasis after ileostomy and post-surgical ischaemia may play a role. In our case, we tested the intestinal barrier function and serum endotoxin level, and the elevated endotoxin level may support bacterial translocation in this scenario. Bacterial overgrowth may also induce an inflammatory response. Unfortunately, we could not collect any stool samples from the patient’s diverted pouch due to the low discharge level. In any subsequent similar scenario, the culture and other tests of stool collected from the ileostomy stoma or diverted pouch will be of great value. Considering the differences in severity and outcome among patients who develop pan-enteritis within one month and beyond one month after surgery, we propose that different pathogeneses may exist. For those who develop pan-enteritis shortly after surgery, faecal stasis may outweigh immune mediation. In this case, improvement of the patient’s situation after closure of the stoma indicates the benefit of re-establishing faecal stasis. However, to verify the above hypothesis, more cases and further mechanistic studies are required.

In conclusion, we report a novel case of severe post-colectomy enteritis; this patient did not respond well to corticosteroid therapy but benefitted from the early closure of the ileostomy and the re-establishment of a natural faecal stream. Because post-colectomy enteritis is a rare condition, which increases the difficulty of conducting large-sample trials, we hope that the experience of our case provides valuable information for future clinical practice.

## Additional file

Additional file 1: Timeline of the case. (DOCX 47 kb)

## Abbreviations

ASCA: Anti-*Saccharomyces cerevisiae* antibody; CMV: Cytomegalovirus.
IPAA: Ileal pouch anal anastomosis
pANCA: Perinuclear antineutrophil cytoplasmic antibody
PUMCH: Peking Union Medical College Hospital
UC: Ulcerative colitis

## References

[1] Haboubi N (2006). Small bowel inflammation in ulcerative colitis. Color Dis.

[2] Corporaal S, Karrenbeld A, van der Linde K, Voskuil JH, Kleibeuker JH, Dijkstra G (2009). Diffuse enteritis after colectomy for ulcerative colitis: two case reports and review of the literature. Eur J Gastroenterol Hepatol.

[3] Valdez R, Appelman HD, Bronner MP, Greenson JK (2000). Diffuse duodenitis associated with ulcerative colitis. Am J Surg Pathol.

[4] Rubenstein J, Sherif A, Appelman H, Chey WD (2004). Ulcerative colitis associated enteritis: is ulcerative colitis always confined to the colon?. J Clin Gastroenterol.

[5] Gooding IR, Springall R, Talbot IC, Silk DB (2008). Idiopathic small-intestinal inflammation after colectomy for ulcerative colitis. Clin Gastroenterol Hepatol.

[6] Feuerstein JD, Shah S, Najarian R, Nagle D, Moss AC (2014). A fatal case of diffuse enteritis after colectomy for ulcerative colitis: a case report and review of the literature. Am J Gastroenterol.

[7] Hoentjen F, Hanauer SB, Hart J, Rubin DT (2013). Long-term treatment of patients with a history of ulcerative colitis who develop gastritis and pan-enteritis after colectomy. J Clin Gastroenterol.

[8] Lin J, McKenna BJ, Appelman HD (2010). Morphologic findings in upper gastrointestinal biopsies of patients with ulcerative colitis: a controlled study. Am J Surg Pathol.

[9] Nakajima M, Nakashima H, Kiyohara K, Sakatoku M, Fujimori H, Terahata S, Nobata H (2008). Case with diffuse duodenitis and enteritis following total colectomy for ulcerative colitis. Nihon Shokakibyo Gakkai Zasshi.

[10] Annese V, Caruso N, Bisceglia M, Lombardi G, Clemente R, Modola G, Tardio B, Villani MR, Andriulli A (1999). Fatal ulcerative panenteritis following colectomy in a patient with ulcerative colitis. Dig Dis Sci.

[11] Uchino M, Ikeuchi H, Bando T, Matsuoka H, Hirata A, Takahashi Y, Takesue Y, Inoue S, Tomita N (2013). Diffuse gastroduodenitis and enteritis associated with ulcerative colitis and concomitant cytomegalovirus reactivation after total colectomy: report of a case. Surg Today.

[12] Terashima S, Hoshino Y, Kanzaki N, Kogure M, Gotoh M (2001). Ulcerative duodenitis accompanying ulcerative colitis. J Clin Gastroenterol.

[13] Rush B, Berger L, Rosenfeld G, Bressler B (2014). Tacrolimus therapy for ulcerative colitis-associated post-colectomy enteritis. ACG Case Rep J.

[14] Rosenfeld GA, Freeman H, Brown M, Steinbrecher UP (2012). Severe and extensive enteritis following colectomy for ulcerative colitis. Can J Gastroenterol.

