# Supplementary material for: A literature review and case report of severe and refractory post-colectomy enteritis
Source: BMC Gastroenterol. 2019 Apr 25;19:61. doi: 10.1186/s12876-019-0974-4 (PMC6482549; doi:10.1186/s12876-019-0974-4)
Supplement: Supplementary file 1 — Timeline of the case. (DOCX 47 kb) [file 12876_2019_974_MOESM1_ESM.docx]

**Timeline of the case**

2016.2

2016.4

2016.5.5

2016.5.14

2016.5.31

2016.8

**closing of ileostomy; after improve,**

**MP tapered down and AZA added**

Symptoms stable, stool volume less than 1L per day;

Endoscopy findings and histological findings improved

**hydrocortisone 50mg q6h i.v.**

**Fluid replacement and NE used to maintain BP, Empirical antibiotics used**

**MP 30mg qd, then tapered**

**Followed-up every 6 months, last follow-up in Mar 2018**

Endoscopy: edematous inflamed mucosa with patchy superficial ulcers;

Pathology: diffuse enteritis

post-colectomy enteritis was diagnosed

Stool volume increased

PE: BP 74/50mmHg, HR 90bpm, poor nutrition status, active bowel sound

Labs: sCr 183μmol/L,

Stool: large number of leukocytes,

Stool cultures and CDAB all negative

Stool volume gradually decreased and gained weight

Stool volume still increased

BP maintained but stool volume also maintained

Urine output decreased

Repeated unconsciousness

PMH: poliomyelitis

2014.12 diarrhea with mucus and blood, diagnosed acute severe UC and backwash ileitis, treated with corticosteroids, refractory and complicated with CMV infection

2015.2 total colectomy and ileostomy, then prednisone was tapered and stopped

2016.1 IPAA
